# Rotational dynamics and dynamical transition of water inside hydrophobic pores of carbon nanotubes

**DOI:** 10.1038/s41598-017-13704-6

**Published:** 2017-11-01

**Authors:** Haruka Kyakuno, Kazuyuki Matsuda, Yusuke Nakai, Ryota Ichimura, Takeshi Saito, Yasumitsu Miyata, Kenji Hata, Yutaka Maniwa

**Affiliations:** 1 Department of Physics, Graduate School of Science and Engineering, Tokyo Metropolitan University, Hachioji, 192-0397 Japan; 2 Institute of Physics, Faculty of Engineering, Kanagawa University, Yokohama, 221-8686 Japan; 3 Nanomaterials Research Institute, National Institute of Advanced Industrial Science and Technology, Tsukuba, 305-8565 Japan; 4 JST, CREST, Kawaguchi, 332-0012 Japan; 5 CNT-application Research Center, National Institute of Advanced Industrial Science and Technology, Tsukuba, 305-8565 Japan

## Abstract

Water in a nanoconfined geometry has attracted great interest from the viewpoint of not only basic science but also nanofluidic applications. Here, the rotational dynamics of water inside single-walled carbon nanotubes (SWCNTs) with mean diameters larger than ca. 1.4 nm were investigated systematically using ^2^H nuclear magnetic resonance spectroscopy with high-purity SWCNTs and molecular dynamics calculations. The results were compared with those for hydrophilic pores. It was found that faster water dynamics could be achieved by increasing the hydrophobicity of the pore walls and decreasing the pore diameters. These results suggest a strategy that paves the way for emerging high-performance filtration/separation devices. Upon cooling below 220 K, it was found that water undergoes a transition from fast to slow dynamics states. These results strongly suggest that the observed transition is linked to a liquid-liquid crossover or transition proposed in a two-liquid states scenario for bulk water.

## Introduction

Water in a nanoconfined geometry exhibits unusual dynamical properties that do not appear in the bulk. Studies of this type of water are useful to design high-performance filtration/separation devices^1–3^ and to reveal the function of hydrophobic biological channels^4–6^. They are also of significant interest with respect to the basic science of water. Water in the bulk has many anomalous properties, such as a density maximum at 277 K. Some scenarios, including the hypothesis of a critical point between two-liquid states present in a deeply super-cooled regime, have been proposed to understand these anomalies^7–9^. However, experimental studies are hampered by a region (150–235 K at ambient pressure) where bulk water inevitably crystallizes. Alternatively, because nanoconfinement suppresses the crystallization of water, many theoretical and experimental studies have been performed in confinement geometries^10–21^, such as water in pores of silica, zeolites, and nanocarbons, and water on protein surfaces. However, a collective view on these properties has not yet been achieved^16–18^. In particular, systematic studies on the effect of pore dimensions, temperature, and the hydrophobicity of material surfaces on water dynamics are rather limited.

In the present study, water inside single-walled carbon nanotubes (SWCNTs) was employed to systematically reveal water dynamics inside “hydrophobic” nanocavities^22^. SWCNTs provide cylindrical pores whose diameter *D* can be systematically controlled from less than 1 nm to a few nanometers. Water inside SWCNTs exhibits several phases upon cooling from room temperature (RT)^23–28^. For thin SWCNTs (*D* < ca. 1.45 nm), liquid water adsorbed at RT is transformed upon cooling into tubule ices with a one-dimensional periodicity, and are referred to as ice-nanotubes (ice-NTs). For thick SWCNTs (*D* > ca. 1.45 nm), on the other hand, water exhibits a wet-dry transition or a hydrophilic–hydrophobic transition at around 220–240 K^22,28^, and the water is partially ejected from the inside of the SWCNTs. However, a substantial quantity of water can be trapped inside the SWCNTs even below the wet-dry transition temperature. Here, this water inside SWCNTs was investigated by nuclear magnetic resonance (NMR) spectroscopy^29,30^ to clarify the water dynamics. It is known that NMR spectroscopy enables the study of large-amplitude molecular motions on the time scale of 10^−5^–10^−11^ s. In water-SWCNTs, the narrowed NMR spectra of water that can be observed even below the bulk freezing temperature have been assigned to liquid or mobile water inside SWCNTs^28,31–37^.

In the present study, we used high-purity SWCNT samples^38–40^ because magnetic impurities present in SWCNT samples, such as residual metal catalysts used in the synthesis of SWCNTs^41^, often prevents their intrinsic properties from being observed by NMR spectroscopy. ^2^H-NMR measurements were performed on heavy water inside SWCNT samples. The results are discussed to evaluate the role of hydrophobicity of the pore walls and pore diameters in the water dynamics and transition, in combination with molecular dynamics (MD) calculations, differential scanning calorimetry (DSC) measurements, X-ray diffraction (XRD) analysis, and data previously reported for hydrophilic pores of MCM-41.

## Results and Discussion

### NMR spin-lattice relaxation time, *T*_1_

**Figure 1:**
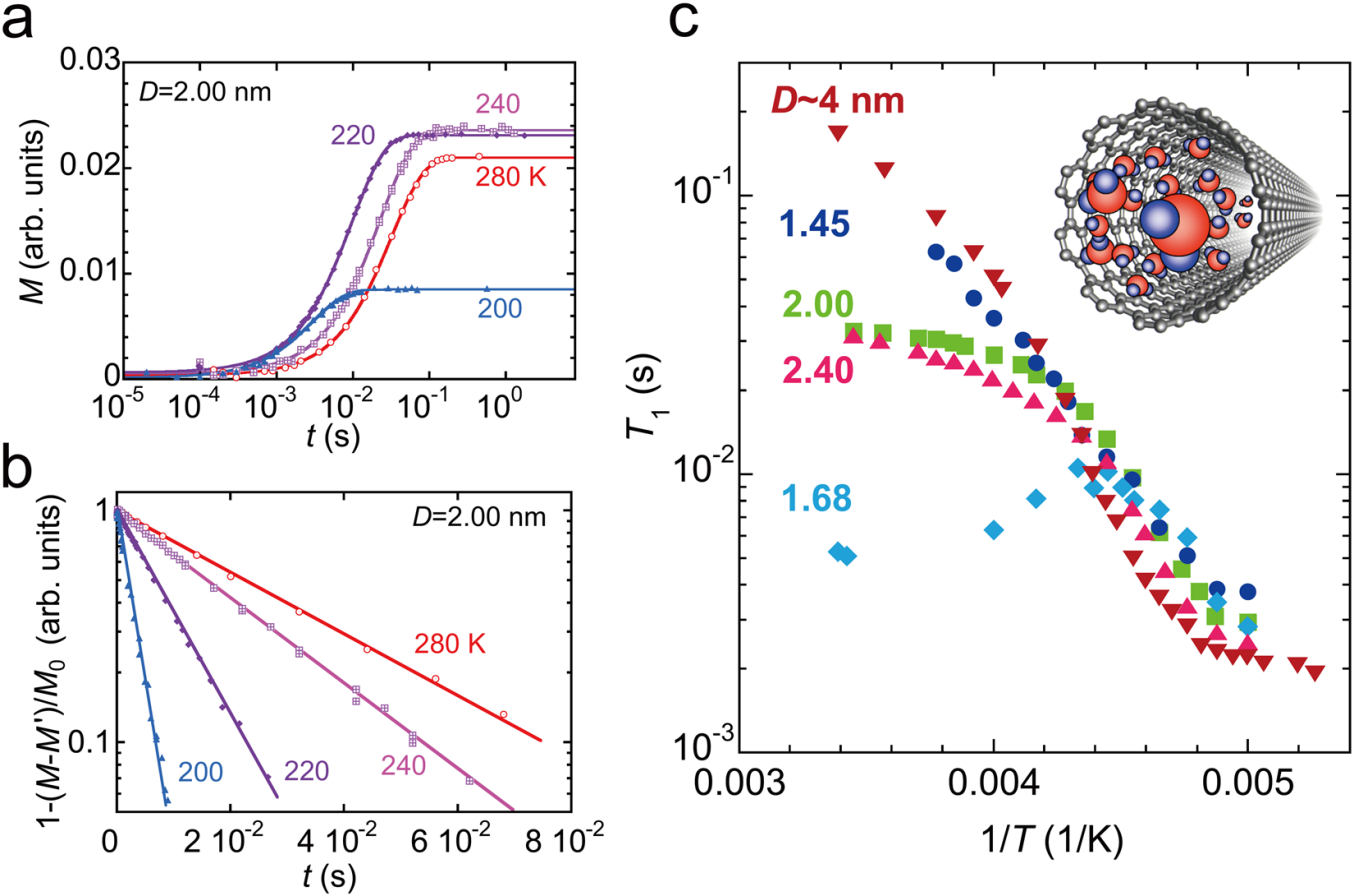
^2^H-NMR results for water-SWCNTs. (**a** and **b**) Relaxation curves measured for the SWCNT sample with a mean diameter of *D* = 2.00 nm. The solid lines are fitted with equation (1). (**c**) Spin-lattice relaxation time *T*_1_, obtained for the SWCNT samples with *D* = 1.45, 1.68, 2.00, 2.40, and 4 nm as a function of $1/T$. The inset shows a schematic illustration of a SWCNT with water molecules. *D* is the mean SWCNT diameter in the sample.

The ^2^H-*T*_1_ of heavy water inside SWCNTs was measured as a function of temperature *T* and SWCNT diameter *D* to probe large-amplitude molecular rotation. Figure 1a shows examples of the raw data acquired to determine *T*_1_ values. NMR signal intensity, *M*(*t*), which is proportional to the nuclear magnetization along the applied static magnetic field, was measured as a function of time *t*, after a saturation comb pulse, and was fitted with equation (1) (in *Methods*) to obtain *T*_1_, *M*′, *β*, and *M*_0_. Figure 1b shows ${\rm{log}}\{1-[M(t)-{M}^{^{\prime} }]/{M}_{0}\}$ as a function of *t*. All the measurements presented here give $\beta \approx 0.95-0.99$, as approximately reproduced by the straight lines for *β* = 1 in Fig. 1b. This implies that *T*_1_ is homogeneous and independent of water sites, or averaged over different water sites inside a SWCNT by the moving or hopping of water molecules within the *T*_1_ process, ca. 10^−3^–10^−1^ s. The small deviation from *β* = 1 may be due to the distribution of SWCNT diameters in the sample because the water dynamics are expected to be somewhat dependent on the SWCNT diameter.

Figure 1c summarizes the *T*-dependences obtained for the different diameter SWCNT samples. At low temperatures, all the samples show a similar *T*-dependence, which can be described by the Bloembergen-Purcell-Pound (BPP) relaxation mechanism^29,30,42^ with the *T*-dependent rotational correlation time of water molecules *τ*_rot_, in equation (2) (in *Methods*). On the other hand, the *T*-dependence in the higher-*T* regions is unusual; the *T*_1_ values do not change systematically with the SWCNT diameter, and these may be correlated to the content of magnetic impurities (see Supplementary Fig. S1). For instance, the *T*_1_ of the magnetically impure samples (*D* = 1.68, 2.00, and 2.40 nm) is substantially shorter than that of the high-purity samples (*D* = 1.45 and 4.0 nm). This suggests that the magnetic impurities dominate the *T*_1_ mechanisms of impure samples in the higher-*T* regions. Note that the observed *T*_1_ may be given by $(1/{T}_{1})={(1/{T}_{1})}_{{\rm{imp}}}+{(1/{T}_{1})}_{{\rm{BPP}}}$, where ${(1/{T}_{1})}_{{\rm{imp}}}$ and ${(1/{T}_{1})}_{{\rm{BPP}}}$ are contributions from magnetic impurities and BPP mechanism, respectively. With lowering temperature, ${(1/{T}_{1})}_{{\rm{BPP}}}$ steeply increases as expected from equation (2) through the strong *T*-dependence of *τ*_rot_. Thus ${(1/{T}_{1})}_{{\rm{BPP}}}$ dominates the observed $1/{T}_{1}$ at low temperatures. Therefore, discussions in the following sections focus on the low-*T* data.

**Figure 2:**
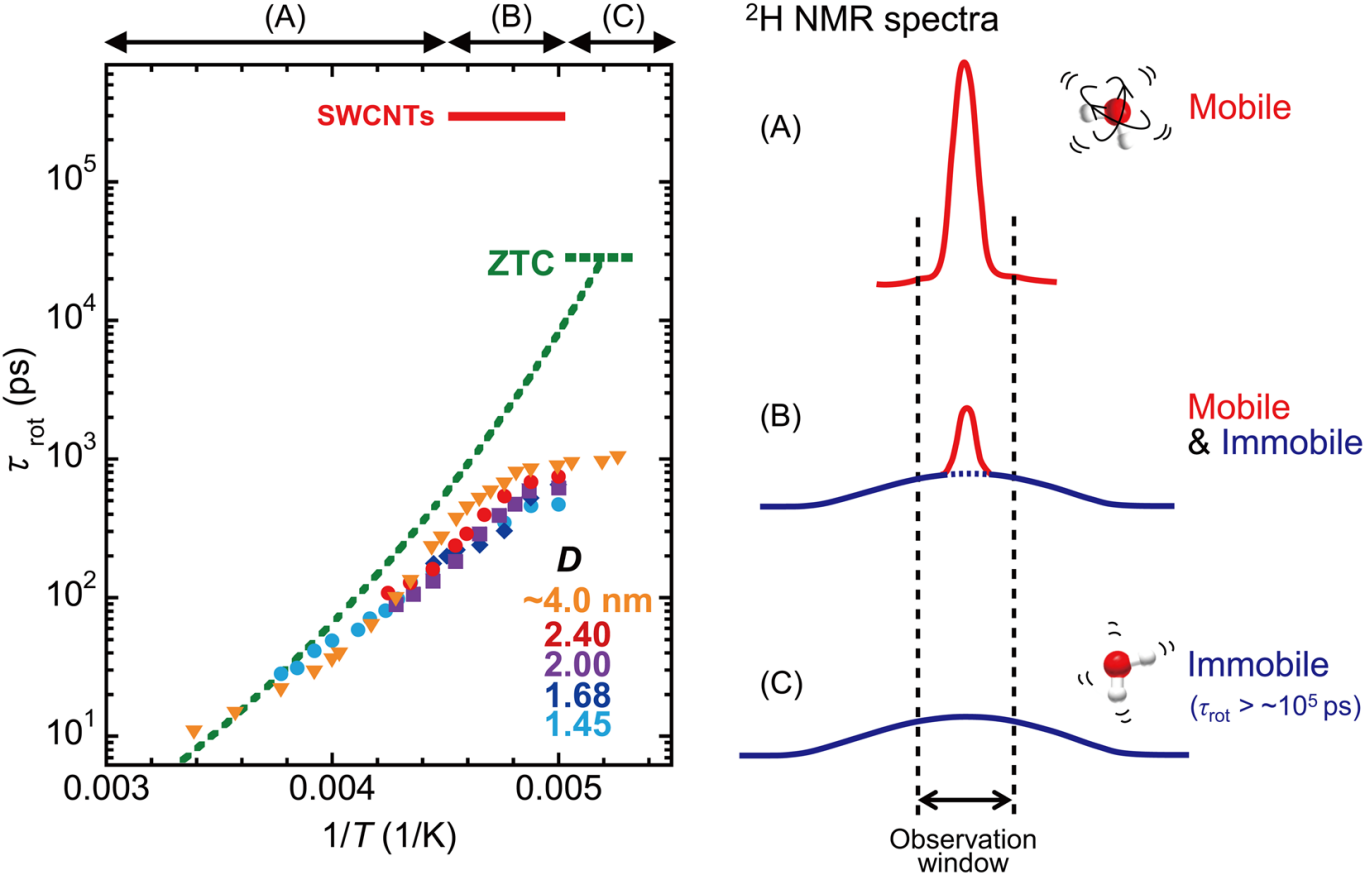
Rotational correlation time (*τ*_rot_) of water molecules inside SWCNTs obtained by ^2^H-NMR. The SWCNT mean diameters are *D* = 1.45, 1.68, 2.00, 2.40, and ~4.0 nm. The dotted line shows the rotational correlation time of water confined inside the three-dimensional pore geometry of zeolite-templated carbon (ZTC) for comparison^45^. The horizontal lines represent the estimated rotational correlation times from the line-broadenings in the SWCNTs and ZTC. The observation window used in ZTC was 2 kHz. Right: Schematic illustrations of the NMR spectra in three temperature regions (**A**), (**B**), and (**C**) shown in the left figure. Only the narrow spectra shown in red are detected in the present measurement setup. Region (**A**): All water molecules have ${\tau }_{{\rm{rot}}} <  \sim {10}^{3}$ ps, and give the narrow NMR spectra. Region (**B**): there are mobile water with ${\tau }_{{\rm{rot}}} <  \sim {10}^{3}$ ps and immobile water with ${\tau }_{{\rm{rot}}} >  \sim {10}^{5}$ ps. Although the present *T*_1_-measurement setup can detect ${\tau }_{{\rm{rot}}} <  \sim {10}^{5}$ ps, *τ*_rot_ of $\sim {10}^{3}- \sim {10}^{5}$ ps was not observed in the present samples. It is strongly suggested that the *τ*_rot_ discontinuously changes in the *T*-region (**B**).

The rotational correlation time, *τ*_rot_, which is estimated from the observed *T*_1_ using equation (2) (in *Methods*) for the BPP relaxation mechanism, is summarized in Fig. 2. The value (*τ*_rot_ = 10–20 ps) extrapolated to 273–300 K is slightly longer than that of bulk water (2–6 ps)^43^, but substantially shorter than a value (ca. 100 ns) reported in a previous NMR study on water inside a SWCNT sample^44^. This discrepancy in the NMR results for water-SWCNTs is probably due to different conditions used for equation (2) to estimate *τ*_rot_. Although equation (2) has two possible solutions for *τ*_rot_ for each *T*_1_ value, the present measurements of *T*-dependence enabled us to unambiguously select the correct value for *τ*_rot_. The discrepancy may also be due to the difference in sample quality used in the experiments.

Upon cooling, *τ*_rot_ increases but it is still shorter than ∼10^3^ ps down to 200 K, where the NMR signal almost disappears due to broadening of the NMR spectra. The *T*-dependence of *τ*_rot_ is described by the Arrhenius *T*-dependence, ${\tau }_{{\rm{rot}}}\propto exp(B/{k}_{B}T)$ with an activation energy *B*, although weak deviations from the Arrhenius law are clearly observed in Fig. 2. It is also found that *τ*_rot_ depends on the SWCNT diameter *D*. With larger *D*, the *T*-dependence of *τ*_rot_ becomes steeper. These features are qualitatively reproduced by MD calculations, which are presented later.

The value ${\tau }_{{\rm{rot}}}\approx 3\times {10}^{5}\,{\rm{ps}}$ is also obtained from the broadening condition of NMR spectra (see *Methods*), as indicated by the horizontal lines in Fig. 2. As shown in Supplementary Fig. S2 and reported in ref.^28^, the NMR signal intensity diminishes at low temperatures and almost disappears at ca. 200 K in all the samples studied. This implies that the NMR spectra were broadened over the instrumental observation windows of ~20 kHz by slowing of the water rotational motion to ${\tau }_{{\rm{rot}}} >  \sim \,3\times {10}^{5}\,{\rm{ps}}$. Therefore, this result indicates that almost all the water transforms into a slow dynamics state at 200 K.

The above estimation for *τ*_rot_ from the NMR linewidth and *T*_1_ indicates that there are two water states with different mobilities at low temperatures: one is mobile water, which gives the narrowed spectra with ${\tau }_{{\rm{rot}}}\approx {10}^{3}$ ps, and the other is an immobile (or less mobile) water, which gives unobservable spectra, with ${\tau }_{{\rm{rot}}} >  \sim \,3\times {10}^{5}{\rm{ps}}$. It is particularly important to realize that there is no evidence for the existence of water molecules with *τ*_rot_ ≈ 10^3^–10^5^ ps over the whole temperature range examined. Therefore, we conclude that the mobile water at high *T* transforms into immobile water upon cooling with a sudden change in *τ*_rot_. This is quite different from the case where water freezes gradually, as in a glass transition. In that case, *τ*_rot_ increases gradually upon cooling, as reported in confined water in zeolite-templated carbon (ZTC) with three-dimensional pores^45^, shown by the dashed line in Fig. 2. The observed features in water-SWCNTs indicate a discontinuous transition between mobile (liquid) water and immobile (solid or less mobile liquid) water.

It is worth noting that the observed two water states are essentially stable within the NMR-*T*_1 _time scale which is larger than 10^−3^ s. Besides, the two types of water can be assumed to form finite-size domains, respectively, so that the observed XRD patterns are reproducible as a superposition of corresponding two distinct XRD patterns (see Supplementary Fig. S5). These features, along with the discontinuous change in water dynamics, are consistent with those of a first-order phase transition. The broad coexisting *T*-region (200–220 K) of mobile and immobile water molecules, observed in the present experiments, could be explained by any inhomogeneities present in the samples, such as distributions of SWCNT diameters and water occupations inside SWCNTs^28^ which can cause a distribution of transition temperature. However, because of lack of convincing evidence for origin of the broad *T*-region, further examinations should be required to rule out other possibilities such as a continuous transition^46^, in which the content ratio of two types of water changes continuously with temperature inside each SWCNT.

### DSC measurements

**Figure 3:**
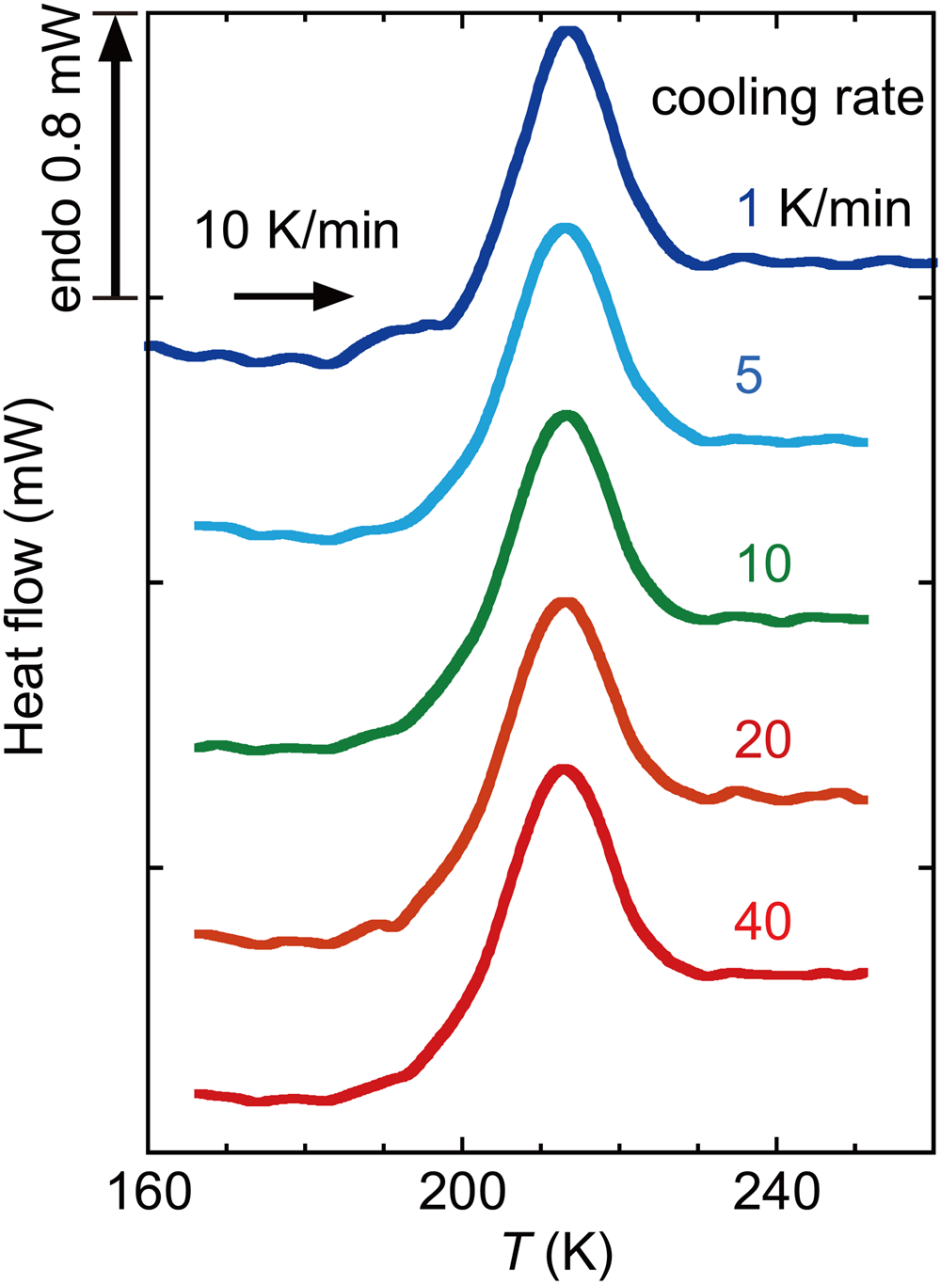
The DSC curves of 2.18 nm SWCNTs as a function of cooling rate. The DSC cell was filled with excess water to inhibit the wet-dry transition.

DSC measurements were performed to characterize the transition observed in the NMR measurements. Figure 3 shows the results for a water-SWCNT sample. After cooling to 130 K at different cooling rates, DSC curves were recorded with heating at 10 K/min, and an endothermic peak was clearly observed around 200–225 K, which suggests a phase transition. Under the present sample conditions, the wet-dry transition was inhibited because the DSC cell was filled with excess water, such that the openings of the SWCNTs were stoppled with bulk ice^22^. Thus, the peak was assigned to the transition of water inside the SWCNTs and not to the wet-dry transition. In addition, no evidence was found for a glass transition because the endothermic peak was not affected by the cooling time.

### MD calculations of *τ*_rot_

**Figure 4:**
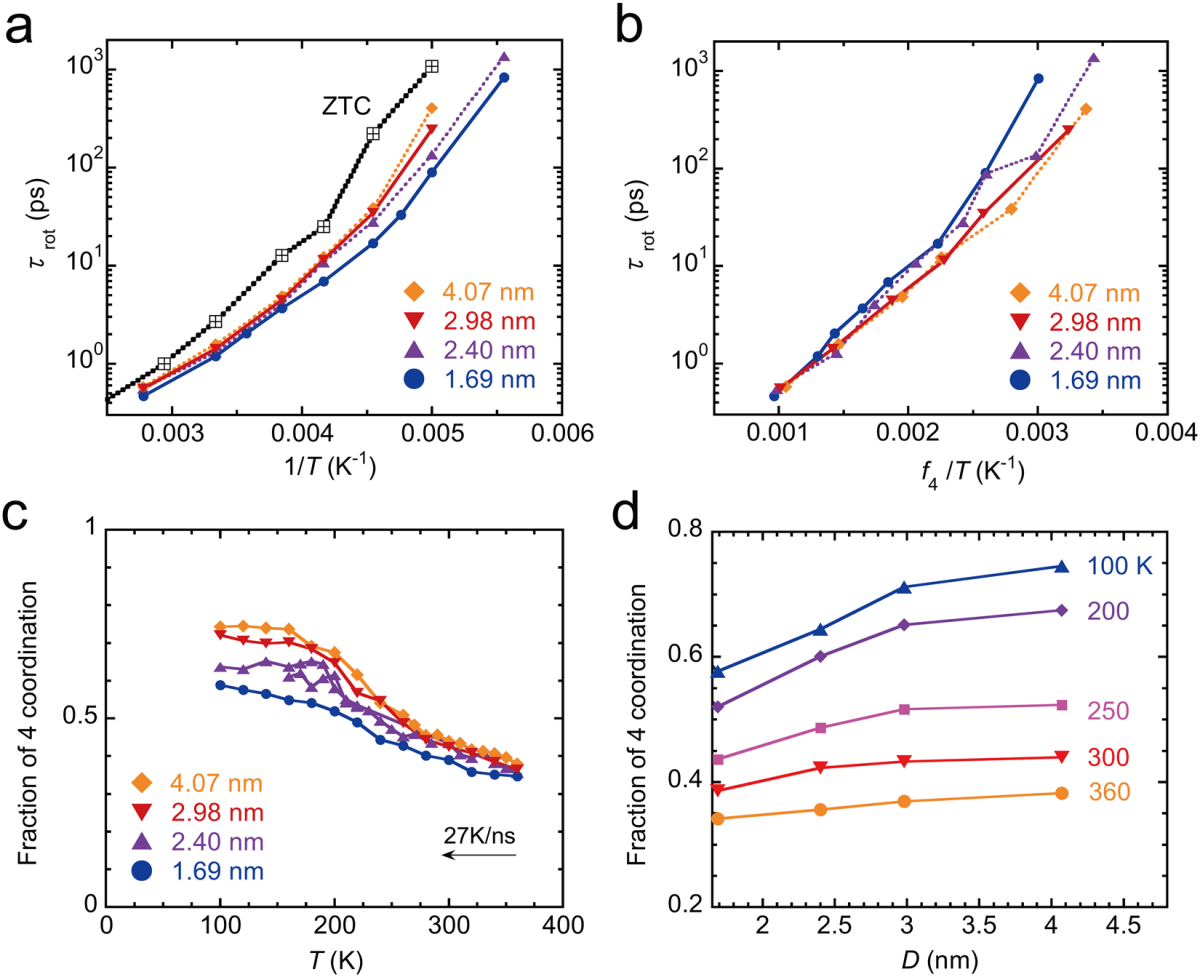
Results of MD calculations for water inside SWCNTs with diameters of 1.69, 2.40, 2.98, and 4.07 nm. (**a**) Calculated rotational correlation time (*τ*_rot_) as a function of *T*. The result for water inside ZTC is also shown for comparison. (**b**) *T*-dependence of *τ*_rot_ as a function of ${f}_{4}/T$. (**c**) and (**d**) Calculated fraction of four coordination water molecules, *f*_4_. Water inside SWCNTs with $D\le 1.41\,{\rm{nm}}$ is transformed into tubular ices (ice NTs) at low-*T* (see Supplementary Fig. S3).

Classical MD calculations were performed to examine the microscopic picture of the molecular dynamics. Figure 4a shows *τ*_rot_ calculated for the SPC/E water^47^ confined in SWCNTs with several diameters, along with that of ZTC^45^. We found the following features from the calculated *τ*_rot_ as functions of *D* and *T*. For SWCNTs with $D\le 1.41$ nm, the water undergoes liquid-solid transitions upon cooling and forms ordered tubular ices (ice NTs) at low temperatures and thus *τ*_rot_ increases steeply below the transition temperature (see Supplementary Fig. S3a). In larger diameter SWCNTs (*D* > 1.6 nm), *τ*_rot_ shows non-Arrhenius *T*-dependence and is better described by a Vogel-Fulcher-Tammann (VFT) form, ${\tau }_{{\rm{rot}}}={\tau }_{0}\,exp[B/(T-{T}_{0})]$. As *D* increases, *τ*_rot_ as a function of $1/T$ becomes steeper. In addition, a comparison between ZTC and SWCNTs suggests that the pore topology plays an important role on the molecular dynamics. The *τ*_rot_ for ZTC with a three-dimensional pore exhibits a stronger *T*-dependence than those of SWCNTs with one-dimensional pores. All these features qualitatively explain the experimental observations as shown in Fig. 2.

Figure 4c,d show the fraction of four-coordination water molecules *f*_4_, where the four-coordination water is defined as a molecule having four neighboring molecules with the O-O distance shorter than 0.33 nm. *f*_4_ can be used as an indicator of the fraction of water molecules that contribute to hydrogen-bonding. *f*_4_ increases for all the SWCNTs with a decrease in *T*, which indicates that a hydrogen-bonding network develops at lower temperatures. For SWCNTs with $D > 1.6$ nm, *f*_4_ increases with *D* (Fig. 4d), which implies that the larger diameter SWCNT has a weaker geometrical restriction for the formation of a hydrogen-bonding network.

The observed non-Arrhenius behavior of *τ*_rot_ may be partially related to the development of such a hydrogen-bonding network with the temperature. As *D* increases, *τ*_rot_ increases along with ${f}_{4}$. Figure 4b shows *τ*_rot_ tentatively plotted as a function of ${f}_{4}/T$. The *T*-dependence at least at high-*T* can be roughly described by ${\tau }_{{\rm{rot}}}={\tau }_{0}exp[{E}_{0}\,{f}_{4}/({k}_{B}T)]$, where ${E}_{0}$ is a constant. This implies that the activation energy, ${E}_{0}\,{f}_{4}$, for the reorientation increases with lowering of the temperature as a result of an increase in ${f}_{4}$ or the development of hydrogen bonds. The value for ${E}_{0}/{k}_{B}$ is in the range of 2500–2900 K and is almost equal to the binding energy for a typical hydrogen bond, 2400 K. The deviation from linearity at low-*T* may be related to the transition observed in the present experiments.

### Role of hydrophobicity

**Figure 5:**
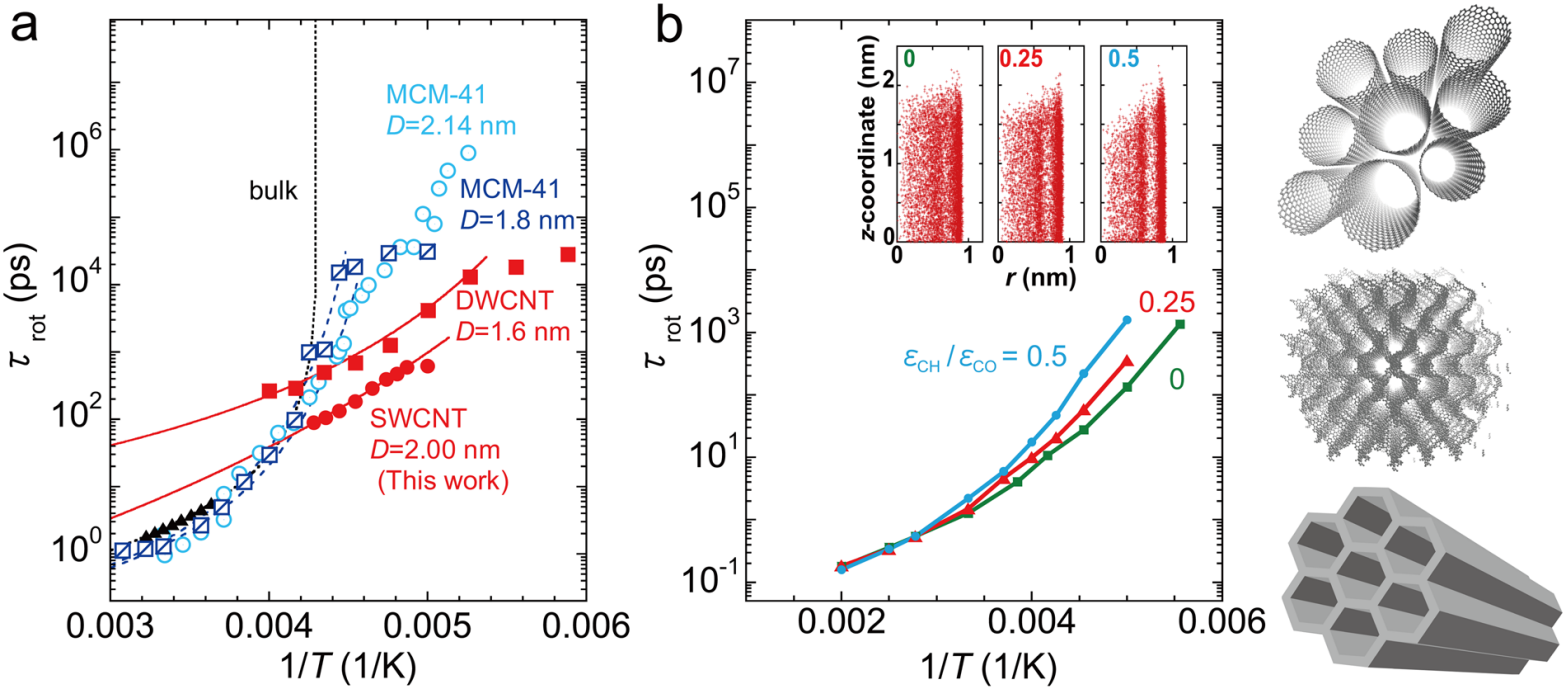
Temperature dependence of correlation times for confined water. (**a**) Comparison of correlation times for water inside SWCNTs, DWCNTs^48^, MCM-41^49,50^, and bulk water^43^. Data represented by circles (MCM-41, *D* = 2.14 nm; SWCNT, *D* = 2.00 nm) and triangles (bulk water) were determined from ^2^H-NMR measurements, while those represented by squares (MCM-41, *D* = 1.8 nm; DWCNT, *D* = 1.6 nm) were from quasielastic neutron scattering (QENS) measurements. The dashed and solid lines are fitted with ${\tau }_{{\rm{rot}}}={\tau }_{0}exp[B/(T-{T}_{0})]$ at high-*T* regions. (**b**) *τ*_rot_ of water inside a 2.00 nm SWCNT as a function of pore hydrophilicity, obtained from MD calculations. The interaction parameters, ${\varepsilon }_{{\rm{CH}}}$, between a carbon atom in a SWCNT and a hydrogen atom in water were taken as ${\varepsilon }_{{\rm{CH}}}/{\varepsilon }_{{\rm{CO}}}=0,\,0.25,\,{\rm{and}}\,0.50$. Insets show the menisci of the confined water molecules at 285 K. *r* is the distance from the symmetry axis or the tube axis of the SWCNT, and *z* is the coordinate along the tube axis. Right: schematic illustrations of a SWCNT bundle, ZTC, and MCM-41 from top to bottom, respectively.

Here, we discuss the effect of pore hydrophobicity on the water dynamics. The large diameter SWCNTs are hydrophobic, particularly at low temperatures^22^; therefore, a comparison of the present results with those for hydrophilic MCM-41 with a similar one-dimensional pore geometry is instructive. The correlation times are compared in Fig. 5a.

It is found from Fig. 5a that the dynamics of the confined water are quite different between CNT systems, including both SWCNT and double-walled carbon nanotubes (DWCNT)^48^, and the MCM-41 systems^49,50^. The water inside CNTs exhibits weaker *T*-dependences than in MCM-41, and has a shorter correlation time at low-*T*, i.e., water is highly mobile at low-*T* in hydrophobic CNTs. The behavior of water in MCM-41 is closer to that of bulk super-cooled water with a singularity around 230 K (see dotted line in Fig. 5a). The effect of hydrophobicity is presumably explained by the different interaction strength between water and the pore walls. In the hydrophobic pores of CNTs, water cannot be bonded to the CNT walls because the dominant interaction between water and carbon atoms in the wall is the van der Waals interaction, which is much weaker than water-water interactions, whereas water molecules are easily bonded or anchored to the silanol groups in the wall of MCM-41. As a result, water inside hydrophobic pores can be more mobile, particularly at low temperatures.

To further support the above discussion, *τ*_rot_ was calculated for SWCNTs with different pore hydrophobicities (Fig. 5b). Here, the hydrophobicity was artificially varied through the use of different interaction parameters between carbon atoms in the SWCNTs and hydrogen atoms in water. This interaction induces water molecules to anchor to the SWCNT wall. As the carbon-hydrogen interaction increases, the meniscus changes toward that observed with hydrophilic pores (see the insets of Fig. 5b), and the *T*-dependence of *τ*_rot_ becomes steeper. This is consistent with the present observations and previous reports^49–51^. Similar tendencies have been reported in previous MD calculations^52,53^.

### Transition temperature, *T*_C_, as a function of SWCNT diameter, *D*

In the range of *T* where the NMR and DSC measurements confirmed the transition, XRD results have suggested a structural transition (see Supplementary Figs S4 and S5, and ref.^22^). An analysis of water diffuse scattering (WDS) patterns arising from water inside SWCNTs provided a consistent result with the NMR measurements, where the WDS patterns can be decomposed into two components corresponding to the higher- and lower-*T* structures (see Supplementary Fig. S5). This coincidence indicates that the dynamical transition inferred from the present NMR study is closely related to the structural change at *T*_C_ observed by XRD.

As shown in Supplementary Fig. S4 and a previous study^22^, WDS patterns change significantly at *T*_C_. Above *T*_C_, the WDS profiles are broad and similar to those for bulk liquid water, whereas they become narrow below *T*_C_. Accordingly, the WDS peak position gradually decreases upon cooling above *T*_C_ toward the lower-*Q* side, and then it shows little change with temperature below *T*_C_. Although these XRD results suggest a structural transition from a liquid state to a solid state, the detailed low-*T* structures have not been identified at present. Multiwalled helical ice structures, which have been suggested to exist inside SWCNTs by computer simulations^26^, are clearly ruled out as the low-*T* structure by a comparison of the observed and calculated XRD patterns (see Supplementary Fig. S6). Hexagonal ice (ice Ih) structures confined inside SWCNTs may be another candidate for the low-*T* structure. However, energetically stable ice Ih structures inside SWCNTs, which are compatible with the observed XRD patterns, have not been obtained at present. The observed patterns are instead rather similar to those reported for MCM-41^54^, suggesting that a disordered structure is a candidate for the low-*T* phase. In combination with the NMR results, the water inside SWCNTs presumably exhibits a transition between a mobile liquid state and a less-mobile disordered (liquid or solid) state at *T*_C_.

**Figure 6:**
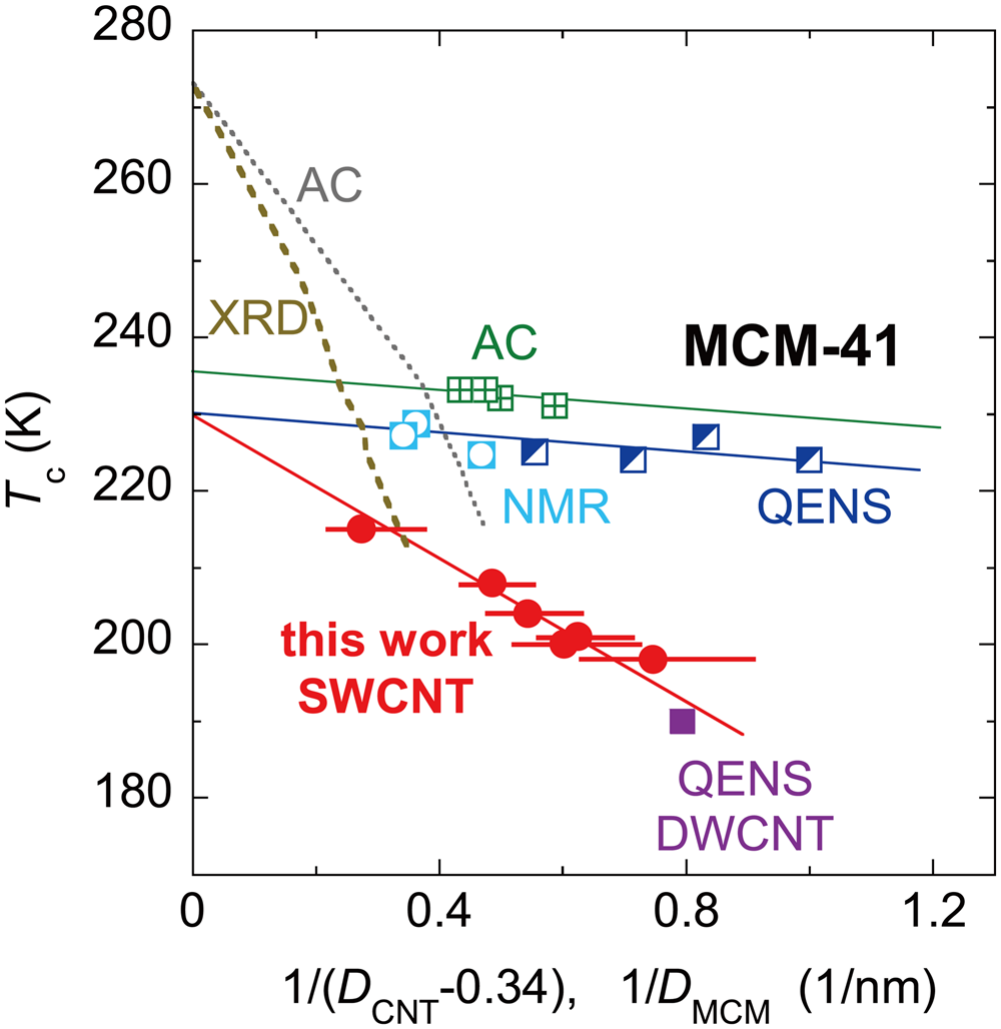
Transition temperature *T*_C_, as a function of pore diameter *D’*, along with distinctive transition temperatures reported previously for water confined in MCM-41. The diameter of the SWCNT pore was defined by ${D}^{^{\prime} }=(D-t)$ where *t* ≈ 0.3 − 0.4 nm, the thickness of the SWCNT wall. AC: melting temperatures of ice Ih (dotted gray line) and heat-capacity maximum temperatures (green squares with crosses) obtained by adiabatic calorimetry for MCM-41^55^; XRD: melting temperatures of ice Ih obtained from XRD patterns of MCM-41^56^; NMR: anomalous temperatures obtained from NMR relaxation times in MCM-41^49^; QENS: anomalous temperatures obtained from quasielastic neutron scattering measurements^48,50^. The closed red circles were obtained from XRD patterns in ref.^22^ and Supplementary Fig. S4 in the present paper.

Next, we discuss the effect of the pore diameter *D*′ on the transition. In SWCNTs, *D*′ is given by ${D}^{^{\prime} }=D-t$ with $t\approx 0.34$ nm as the thickness of the SWCNT wall (see *Methods*). Figure 6 plots the *T*_C_ as a function of $1/{D}^{{\rm{^{\prime} }}}$, along with several distinctive transitions or anomalous temperatures reported for water adsorbed inside MCM-41^49,50,55,56^ and DWCNTs^48^. The *T*_C_ is nearly proportional to $1/{D}^{{\rm{^{\prime} }}}$, as shown by the straight lines in Fig. 6, similar to the depression of the melting temperature of ice Ih (see thick and thin dashed lines in Fig. 6)^55,56^. When the pore diameter is extrapolated to that of the bulk ($1/{D}^{{\rm{^{\prime} }}}\to 0$), the *T*_C_ for the CNT system, as well as the anomalous temperatures for the MCM-41 system, is ca. 230 K. Interestingly, this is almost equal to a hypothetical singularity temperature of ${T}_{{\rm{s}}}\approx 228$ K for bulk water^7–9^, rather than the melting temperature (*T*_*m*_ = 273 K) of ice Ih. This strongly suggests that the nanoconfinement depresses the bulk hypothetical singularity temperature to *T*_C_. The melting (freezing) temperature of ice Ih, if present, may be depressed below *T*_C_ in the diameter range of the present SWCNT samples.

By comparing the results for the hydrophobic CNTs and hydrophilic MCM-41 systems, it was found that the depression of *T*_*s*_ by nanoconfinement is much stronger in the CNT system than in the MCM-41 system. In addition, it is worth noting that the discontinuous transition was found at *T*_c_ in the present SWCNTs while continuous changes, which may be ascribed to a liquid-liquid crossover (or the Widom line), have been reported in the MCM-41 system^50^. Therefore, it is strongly suggested that the hydrophobicity of pore walls plays an important role in the transition behaviors of confined water, as well as the rotational dynamics. This would be caused through differences in the surface energy between the pore wall and water.

All the above discussions strongly support a scenario that there are two liquid states in water^7–9^. When the temperature passes through the *T*_s_ upon cooling, the bulk super-cooled liquid water is transformed into another liquid state (referred to as low density liquid). Upon further cooling, this liquid is presumed to freeze into an amorphous ice (referred to as low density amorphous ice). The present study strongly suggests that the transition between these two liquid states is directly revealed in the nanoconfined geometry.

## Conclusions

Confined water inside hydrophobic SWCNTs was studied using ^2^H-NMR, DSC, XRD measurements, and MD calculations in the temperature range of 300–150 K for SWCNT diameters in the range of 1.45–4.0 nm. Using high-purity SWCNTs, intrinsic information on water dynamics was successfully obtained. It was demonstrated that fast molecular rotation continues until ca. 220 K, and the *T*-dependence is much weaker than that in the hydrophilic pores of MCM-41, three-dimensional pores of ZTC, and bulk water. The observed rotational dynamics were well reproduced by MD calculations. Upon cooling below 220 K, water inside the SWCNTs undergoes a transition at *T*_c_, which is dependent on *D*. The lower-*T* state is presumably a disordered solid (or liquid) with a rotational correlation time larger than 10^−7^ s. At the limit to the bulk water ($1/D\to 0)$, the *T*_c_ is extrapolated to ca. 230 K, the singularity temperature of super-cooled bulk water, which suggests that the nanoconfinement by hydrophobic pores reduces the bulk singularity temperature and leads to a discontinuous dynamical transition. This observation is favorable for the liquid-liquid transition scenario of bulk water^7–9^. The present study established that the hydrophobicity of pore walls and the pore dimensionality have a significant effect on the molecular dynamics of confined water. The results provide information that is helpful not only for the design of high performance nanofluidic devices but also for further studies into the unsolved properties of bulk water.

## Methods

### Characterization of SWCNT samples

Experiments were performed on SWCNT samples with mean diameters *D* = 1.45 ± 0.07, 1.68 ± 0.25, 2.00 ± 0.29, 2.40 ± 0.26 nm^22^, and 4 nm. The 4 nm SWCNT sample had a diameter distribution of 3–5 nm^38^. The samples with *D* = 1.68, 2.00, and 2.40 nm were synthesized using the enhanced direct-injection pyrolytic synthesis (eDIPS) method without further purification^39,40^. A previous study involving resonance Raman scattering measurements indicated that SWCNT samples synthesized by the eDIPS method contained relatively few amorphous carbon impurities and few structural defects. The 1.45 nm SWCNT sample (ArcSo grade) was purchased from Meijyo Nanocarbon Ltd., and it was highly purified by a previously reported method^41^. The 4 nm SWCNT sample was synthesized by the super growth (SG) method^38^. Powder XRD patterns for these samples were reported in a previous paper^22^ and that for the 4 nm SWCNT sample is given in Supplementary Fig. S4. Magnetic susceptibility measurements were conducted using a superconducting quantum interference device (SQUID) magnetometer (MPMS, Quantum Design) to detect magnetic impurities present in the samples, and the results for the 1.45 and 4 nm samples and a typical eDIPS sample are shown in Supplementary Fig. S1. Compared to the 4 nm SG and purified 1.45 nm samples, the eDIPS samples had larger contents of magnetic impurities.

The raw SWCNTs had few openings (i.e., structural defects) for the introduction of water into the pores; therefore, they were heat-treated in air to open their walls^28^. We presume that these openings are stable at least below RT. The SWCNT samples were sealed in a quartz tube saturated with water vapor after vacuum-pumping down to ca. 1 Pa, except for DSC measurements. The details of this procedure have been reported in previous papers^22,28,33^. The amount of water adsorbed inside the opened SWCNTs with *D* = 1.4–2.4 nm was estimated to be 20–57 wt%, with respect to the dry SWCNTs (see Figs S2–S4 in the Supplementary Material of ref.^22^). The SWCNT diameter *D* is defined by the centers of carbon atoms in a SWCNT, which implies that the effective pore diameter is given by $D-t$ where $t\approx 0.34$ nm, the thickness of the SWCNT wall.

### NMR measurements

A pulsed Fourier transform NMR technique was used to obtain NMR signals from heavy water in the *T*-range of 150–300 K. The external magnetic field was 4.0 T, which corresponds to the NMR frequency of $\omega /2\pi =26.22$ MHz, with a field inhomogeneity of 2–4 ppm over the sample volume. ^2^H-NMR spectra for water-SWCNTs with *D* = 1.4–2.4 nm have been reported previously^28^. The spectra of the 4 nm SWCNT sample are presented in Supplementary Fig. S2.

In the present study, spin-lattice relaxation times *T*_1_ for deuterons (^2^H) were used. *T*_1_ relaxation is caused by fluctuating fields at the nuclear sites that are generated by water dynamics^29,30^, and the observed time scale easily extends shorter than 10^−11^ s. The *T*_1_ was measured using a saturation recovery method. The recovered nuclear magnetization measured at a time *t* after the saturation pulses is fitted by a stretched exponential function:

1 $$M(t)={M}_{0}\{1-exp[-{(t/{T}_{1})}^{\beta }]\}+{M}^{^{\prime} },$$

where *M*′ is an instrumental offset. Here, the NMR intensity or ${M}_{0}$ is inversely proportional to the absolute temperature *T* and the number of nuclei of interest because the magnitude of nuclear magnetization is well approximated by the Curie law.

To obtain information on molecular dynamics, the Bloembergen-Purcell-Pound (BPP) relaxation mechanism^42^ was assumed. Here, the spin-lattice relaxation time is dominated by the quadrupole interaction of ^2^H nuclei modulated by the molecular reorientations in heavy water because the quadrupole interaction is much larger than other interactions, such as the intra- and inter-molecular dipole-dipole interactions and chemical shielding interactions. Assuming the BPP mechanism, the observed *T*_1_ is given by:

2 $$\frac{1}{{T}_{1}}=\frac{3}{40}{(\frac{{e}^{2}qQ}{\hslash })}^{2}(1+\frac{{\eta }^{2}}{3})[\frac{{\tau }_{{\rm{rot}}}}{1+{(\omega {\tau }_{{\rm{rot}}})}^{2}}+\frac{4{\tau }_{{\rm{rot}}}}{1+4{(\omega {\tau }_{{\rm{rot}}})}^{2}}],$$

where the quadrupole coupling constant of *e* ^2^ *qQ*/*h* = 195 kHz and its asymmetric parameter of $\eta =0.1$ are used for heavy water molecules. *τ*_rot_ is the rotational correlation time of water molecules. The translational diffusion has only a small contribution to the relaxation because the quadrupole coupling interaction is approximately determined by the intramolecular nature of water. However, if magnetic impurities are present in the sample, they can contribute significantly to the relaxation.

The motion of water molecules is also obtained from the phenomena of motional narrowing of the NMR spectrum. When the motion of water molecules is sufficiently slow or static, the NMR spectrum of water in polycrystalline or glassy samples is broadened by nuclear dipole-dipole interactions, electrical quadrupole interactions (for heavy water), and interactions with magnetic impurities when present in the samples. This is because these interactions generate a magnetic field distribution to which nuclear species of interest are subjected. However, once fast and large amplitude molecular motions start, the NMR spectrum is narrowed because the inhomogeneous field distribution is time-averaged away. This is known as motional narrowing of the NMR spectra. In the case of ^2^H-NMR measurements with heavy water, this occurs when the time scale of molecular rotational motion typically becomes shorter than 10^−6^ s.

Furthermore, if the instrumental window Δ*W* to observe the NMR signal is smaller than the width of the NMR spectrum, the observed signal intensity becomes weaker depending on the Δ*W*. This occurs at $({\tau }_{{\rm{rot}}}2\pi \Delta f)\Delta f \sim \Delta W$ where Δ*f* is the width of the static limit, using the extreme narrowing condition. Setting Δ*f* ∼ 100 kHz and Δ*W* ∼ 20 kHz in the present measurements, we obtained ${\tau }_{{\rm{rot}}} \sim 3\times {10}^{-7}{\rm{s}}=3\times {10}^{5}\,{\rm{ps}}$. The NMR signal of bulk water, which may exist outside SWCNTs, disappears below 273 K (or 250 K under super-cooling) because $\Delta f\gg \Delta W$ and ${\tau }_{{\rm{rot}}}\gg 3\times {10}^{5}\,{\rm{ps}}$. In the present study, thus, it is assumed that the low temperature NMR signal is dominated by liquid-like water confined inside the SWCNTs as reported previously^28,32,36^. Note that the NMR intensity or ${M}_{0}$ in equation (1) is inversely proportional to the absolute temperature *T* because the nuclear magnetization is well approximated by the Curie law. In a similar way, the NMR signal of water inside the thin SWCNTs (1.4 nm SWCNT sample) disappears at low temperatures due to spectral broadening because the water is transformed into ice-NTs or filled ice-NTs^32^.

### DSC measurements

DSC measurements were conducted using a calorimeter (DSC 60 Shimadzu Ltd.) in the range of 130–300 K as a function of water content. A typical amount of SWCNT samples was ca. 4 mg, which was sealed inside an aluminum cell with ultrapure water (H_2_O). The measurements were conducted under a dry air atmosphere of 0.1 MPa.

### MD simulations

The detailed microscopic structure and dynamics of water inside SWCNTs was studied by classical MD simulations using SIGRESS ME 2.3 (Fujitsu Ltd.) for the SPC/E water model^47^. Finite length SWCNTs with diameters of 4.068, 2.983, 2.400, 1.694, 1.411, 1.317, and 1.153 nm, and lengths in the range of 7.166–25.10 nm were investigated. Chiralities of these SWCNTs are (30, 30), (22, 22), (22, 13), (18, 6), (15, 5), (13, 6), and (9, 8), respectively. Calculations were performed in NVT ensembles. The carbon atoms making up the SWCNTs were fixed in the simulation cells and no periodic boundary condition was applied. The potential between the SPC/E water and carbon atoms in SWCNTs was described by the 12–6 Lennard-Jones (LJ) potential:$\,{U}_{{\rm{CO}}}(r)=4{\varepsilon }_{{\rm{CO}}}[{({\sigma }_{{\rm{CO}}}/r)}^{12}-{({\sigma }_{{\rm{CO}}}/r)}^{6}]$ with parameters of ${\varepsilon }_{{\rm{CO}}}/{k}_{{\rm{B}}}=46.88\,{\rm{K}}$ and ${\sigma }_{{\rm{CO}}}=0.3285$ nm, where ${k}_{{\rm{B}}}$ is the Boltzmann constant. Here, *r* is the distance between a carbon atom in a SWCNT and an oxygen atom in water.

The system temperature was gradually decreased from 360 K, where water molecules were highly mobile, down to 100 K with a typical rate of 25–50 K/ns. From the MD results, snapshot structures at each temperature were extracted and they were equilibrated at the temperature for more than 2 ns to calculate rotational correlation functions for the SPC/E water. However, because the correlation time becomes longer with a decrease in the temperature, the water system could not be equilibrated at low temperatures, ca. below 200 K.

The rotational correlation functions ${C}_{\ell }(t)=\langle {P}_{\ell }[\,cos\,\theta (t)]{P}_{\ell }[\,cos\,\theta (0)]\rangle$, where ${P}_{\ell }[\,cos\,\theta (t)]$ denotes a Legendre polynomial of rank $\ell$, were calculated from the MD results. Here, *C*_2_(*t*) can be obtained from NMR and light scattering^57^, while ${C}_{1}(t)$ is obtained from dielectric spectroscopy. Therefore, ${C}_{2}(t)$ was compared with the NMR results:

3 $${C}_{2}(t)=\frac{1}{2}\langle 3{cos}^{2}\theta (t)-1\rangle =\frac{1}{2}\langle 3{\{{\vec{u}}_{i}(t)\cdot {\vec{u}}_{i}(0)\}}^{2}-1\rangle ,$$

where ${\vec{u}}_{i}(t)$ is the unit vector of the molecular axis, and $\theta (t)$ is the angle between ${\vec{u}}_{i}(t)$ and ${\vec{u}}_{i}(0)$. When the rotational correlation function decays non-exponentially with time, it is often described by the Kohlrausch expression:

4 $${C}_{\ell }(t)\propto exp[-{(t/{\tau }_{\ell })}^{{\beta }_{\ell }}],\,{\beta }_{\ell }\le 1.$$

In the present case, it was well reproduced with the fractional exponent, ${\beta }_{\ell }=0.5-1.0$.

### XRD experiments

Powder XRD measurements of dry and wet SWCNT samples were performed using synchrotron radiation X-rays with a wavelength of $\lambda =0.100$ nm at the BL8B station of the Photon Factory, High Energy Accelerator Research Organization (KEK), Japan. The diffracted X-rays were recorded with an imaging plate. The resolution for the scattering angle $2\theta$, of the diffracted X-rays was 0.03°. For a wet sample, the SWCNT films were sealed inside a XRD quartz capillary with saturated water (H_2_O) vapor of ultrahigh purity at RT. The dry sample was sealed after it was evacuated. The amplitude of the X-ray scattering wave vector is defined by $Q=4\pi \,sin\,\theta /\lambda$. The sample temperature was controlled by a gas-blow type cryostat (Rigaku) in the temperature range of 100–350 K. XRD studies for 1.4–2.40 nm SWCNT-water samples have been reported previously^22,28^. The results for the 4 nm SWCNT sample is shown in Supplementary Fig. S4.

### Data availability

All data generated or analysed during this study are included in this published article and its Supplementary Information files.

## Electronic supplementary material

Supplementary Information
