# Supplementary material for: Rotational dynamics and dynamical transition of water inside hydrophobic pores of carbon nanotubes
Source: Sci Rep. 2017 Nov 1;7:14834. doi: 10.1038/s41598-017-13704-6 (PMC5666012; doi:10.1038/s41598-017-13704-6)
Supplement: Supplementary file 1 — Supplementary Information [file 41598_2017_13704_MOESM1_ESM.pdf]

Supplementary Information

Rotational dynamics and dynamical transition of water inside hydrophobic pores of carbon nanotubes

Haruka Kyakuno[1,2,*], Kazuyuki Matsuda[2], Yusuke Nakai[1], Ryota Ichimura[1], Takeshi Saito[3], Yasumitsu Miyata[1,4], Kenji Hata[5] & Yutaka Maniwa[1,*]

[1]*Department of Physics, Graduate School of Science and Engineering, Tokyo Metropolitan University, Hachioji 192-0397, Japan*
[2]*Institute of Physics, Faculty of Engineering, Kanagawa University, Yokohama 221-8686, Japan*
[3]*Nanomaterials Research Institute, National Institute of Advanced Industrial Science and Technology, Tsukuba 305-8565, Japan*
[4]*JST, CREST, Kawaguchi 332-0012, Japan*
[5]*CNT-application Research Center, National Institute of Advanced Industrial Science and Technology, Tsukuba 305-8565, Japan*

[*]Corresponding authors:
H. Kyakuno: h-kyakuno@kanagawa-u.ac.jp
Y. Maniwa: maniwa@phys.se.tmu.ac.jp

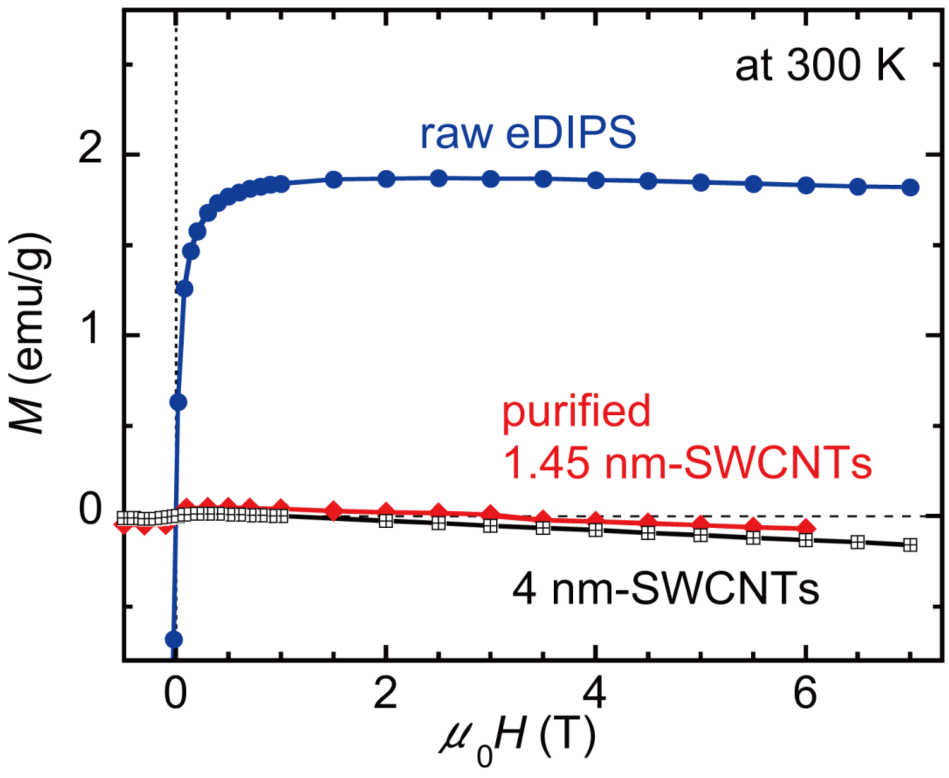

Fig. S1. Magnetization measurements of a typical non-purified eDIPS sample, a purified 1.45 nm SWCNT sample and a 4 nm SWCNT sample. The steep increases below 1 T are due to ferromagnetic impurities, while the linear dependences above ca. 2 T are due to the intrinsic diamagnetic moment of SWCNTs[1].

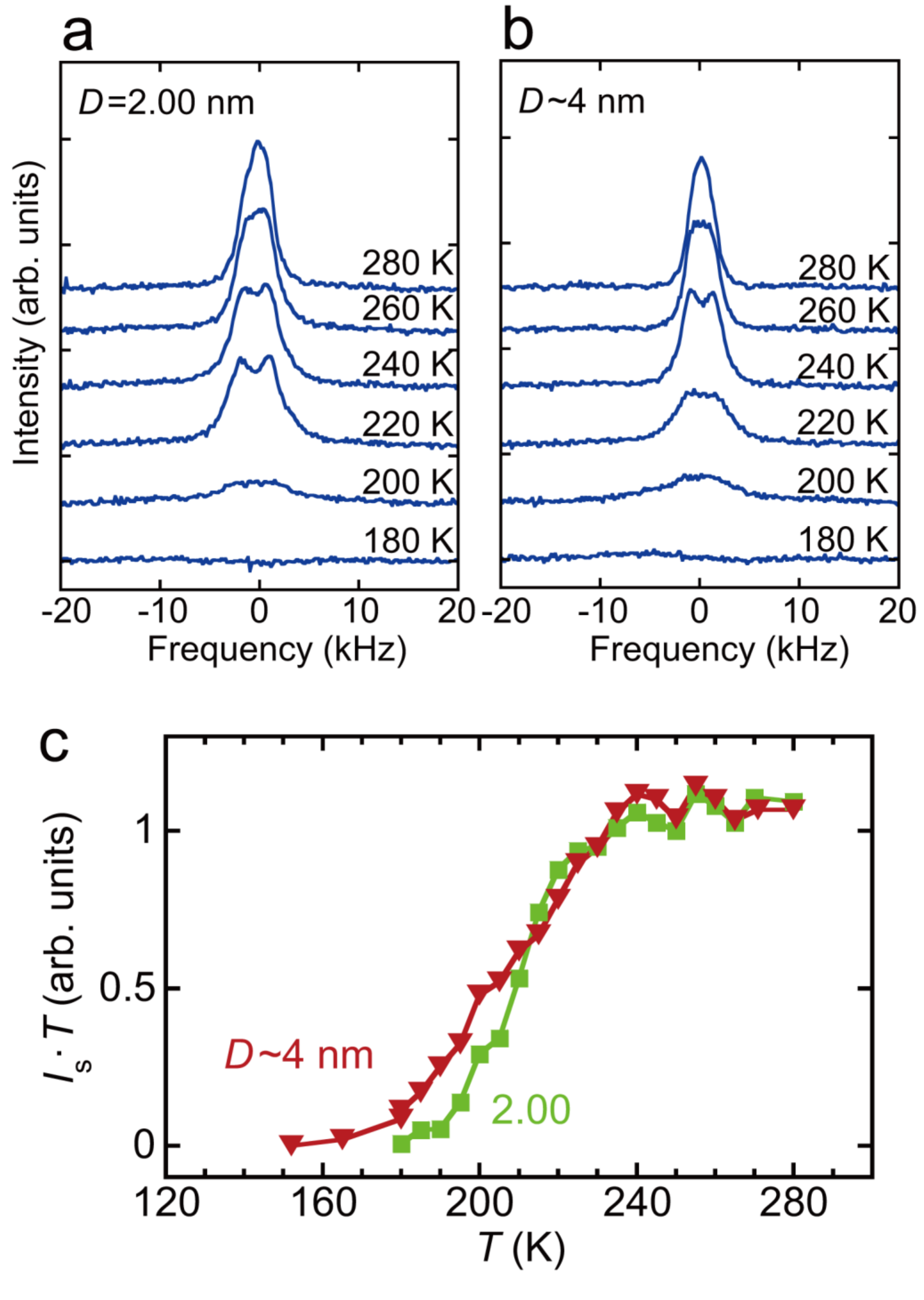

Fig. S2. ^{2}H-NMR results for water inside the 2.00 nm and 4 nm SWCNT samples. **(a)** and **(b)** NMR spectra at several temperatures for 2.00 nm and 4 nm SWCNT samples, respectively. **(c)** Temperature dependence of the integrated NMR intensity multiplied by temperature, which is proportional to the amount of mobile water in the samples.

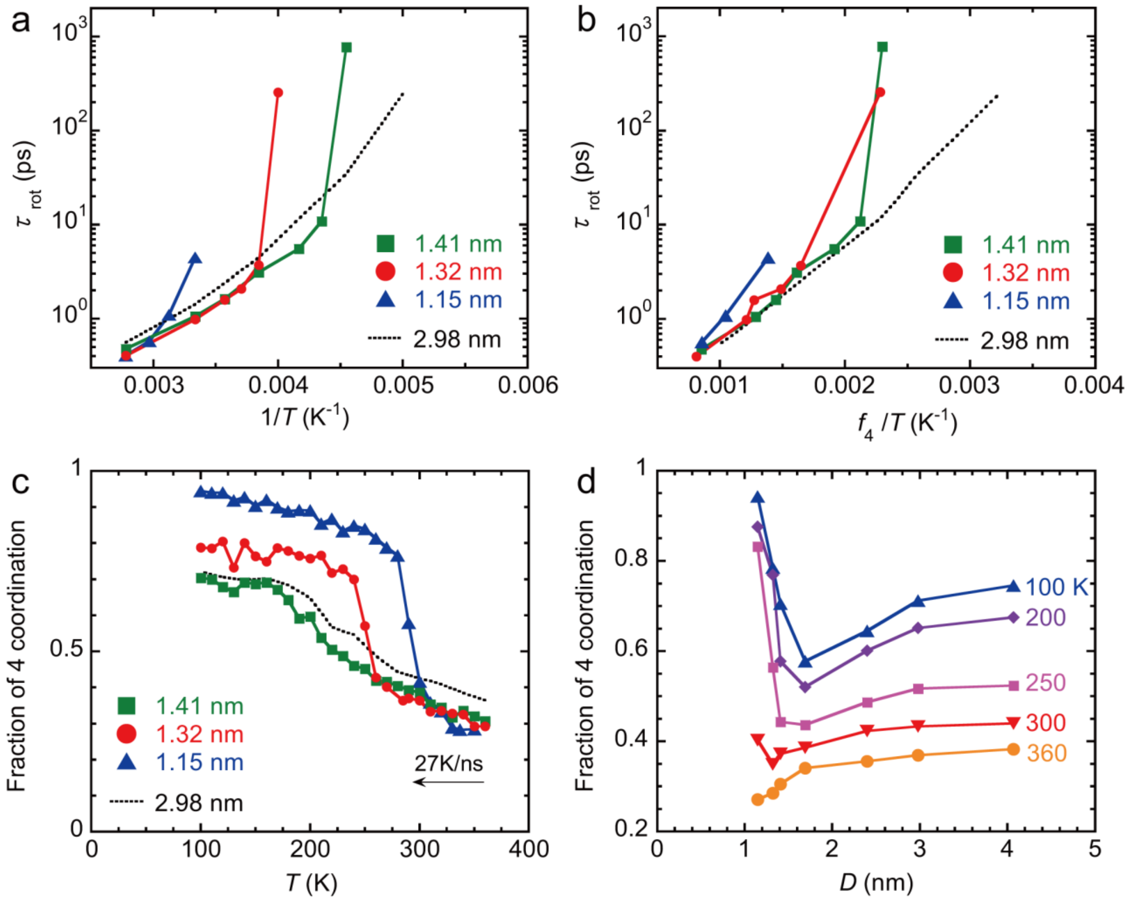

Fig. S3. Results of MD calculations for water inside SWCNTs with diameters of 1.15 – 1.41 nm. (**a**) Calculated rotational correlation time (τ_{rot}) as a function of T. (**b**) T-dependence of τ_{rot} as a function of f_4/T. (**c**) and (**d**) Calculated fraction of four coordination water molecules, f_4. Water inside SWCNTs with $D \leq 1.41$ nm is transformed into tubular ices (ice NTs) at low-T.

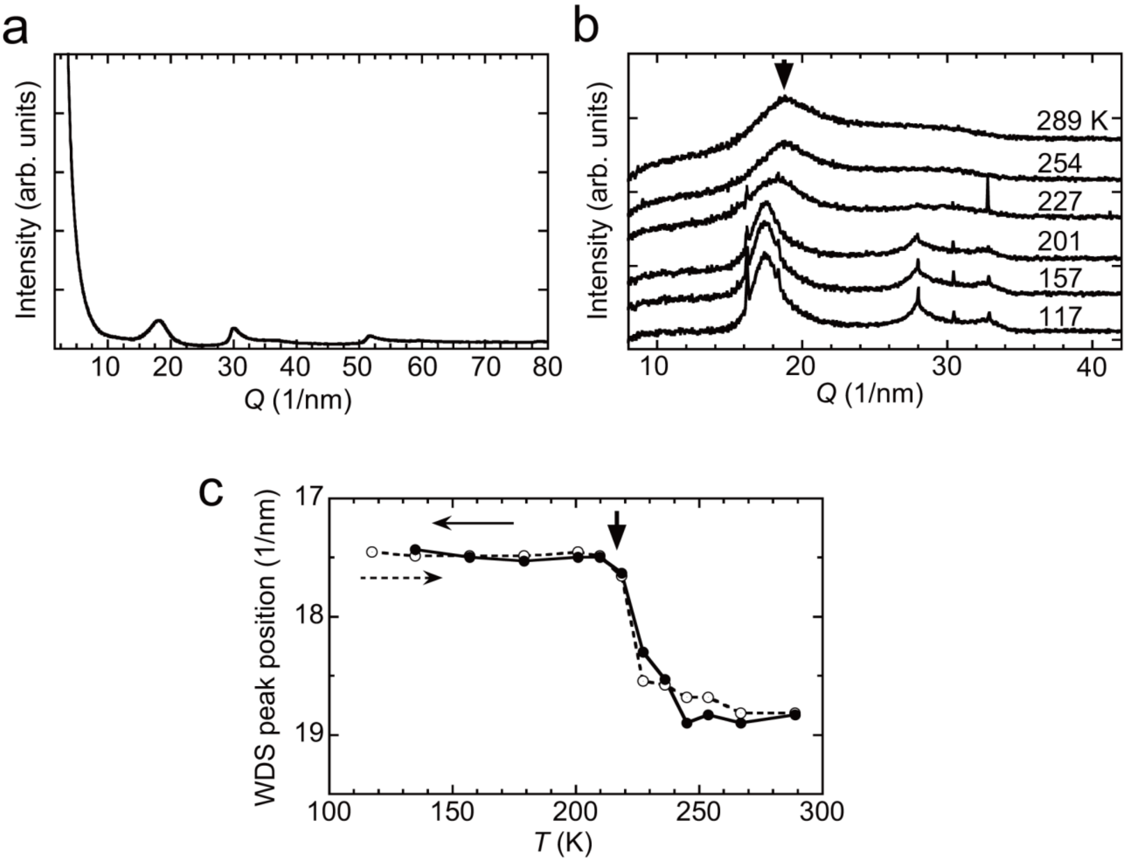

Fig. S4. XRD patterns of 4 nm SWCNT samples. (**a**) XRD pattern of a dry sample. Peaks from SWCNT bundles cannot be observed because of the large average diameter *D* (ca. >3.2 nm) of the SWCNTs, which is consistent with data presented at the website of Zeon Nanotechnology Co., Ltd. (http://www.zeonnanotech.jp/en/products.html). **(b)** XRD patterns of the water-SWCNTs at several temperatures in the water diffuse scattering (WDS) region[2]. **(c)** Temperature dependence of WDS peak position denoted by the arrow in **(b)**. The thick arrow indicates a structural transition temperature.

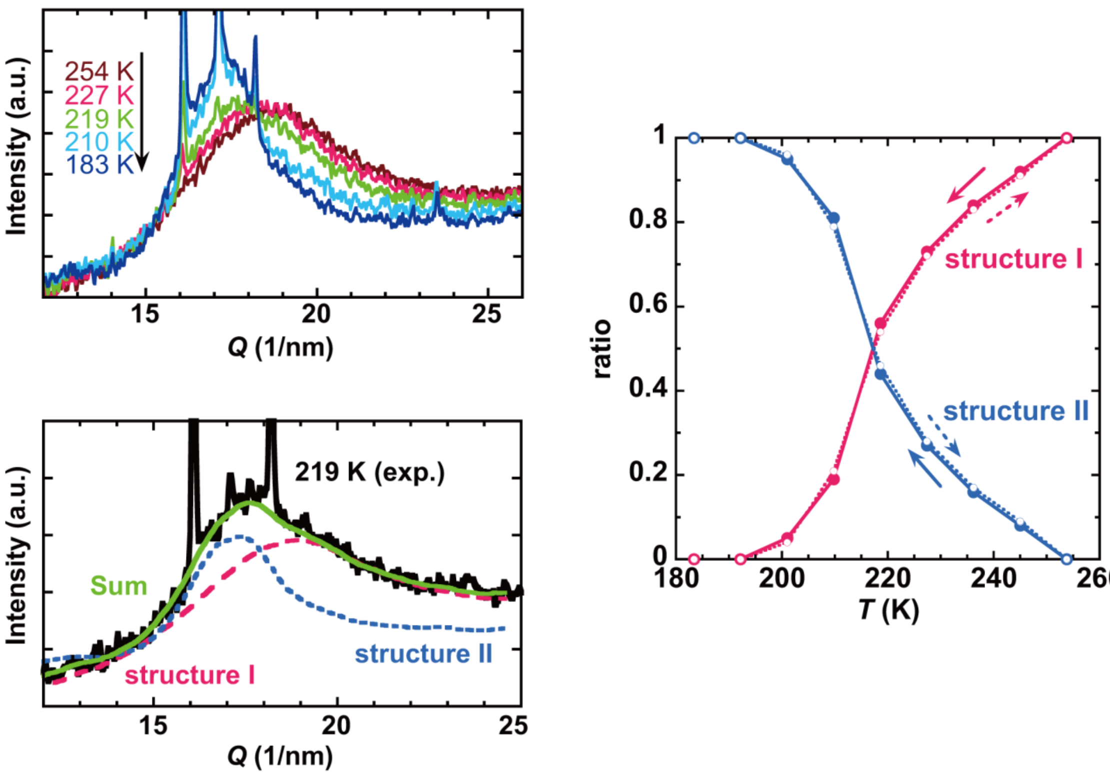

Fig. S5. Analysis of XRD patterns of water inside the 2.40 nm SWCNT sample. The left top figure shows the observed XRD patterns at several temperatures. As in the left bottom figure, it is found that each pattern can be decomposed into two XRD patterns of higher-T structure (structure I) and lower-T structure (structure II), where the XRD pattern at 254 K (183 K) was taken as that of structure I (structure II). The relative intensity of structure I and structure II is given in the right figure. The results suggest that the structural transition occurs discontinuously. Similar XRD analysis has been applied to demonstrate a first-order transition of water in protein crystals[3].

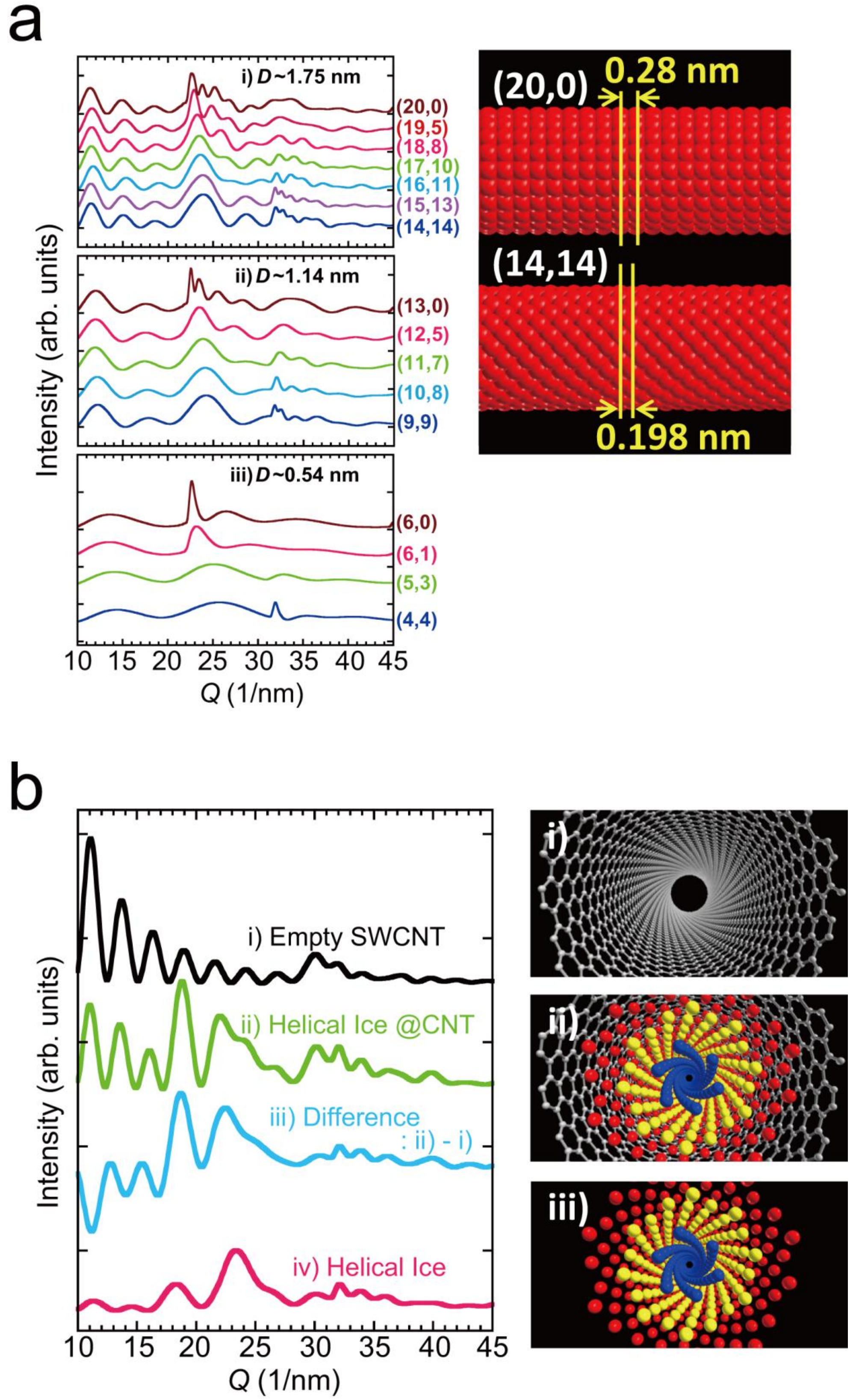
a
i) D~1.75 nm
(20,0)
(19,5)
(18,8)
(17,10)
(16,11)
(15,13)
(14,14)
ii) D~1.14 nm
(13,0)
(12,5)
(11,7)
(10,8)
(9,9)
iii) D~0.54 nm
(6,0)
(6,1)
(5,3)
(4,4)
Intensity (arb. units)
10 15 20 25 30 35 40 45
Q (1/nm)
(20,0)
0.28 nm
(14,14)
0.198 nm
b
i) Empty SWCNT
ii) Helical Ice @CNT
iii) Difference
: ii) - i)
iv) Helical Ice
Intensity (arb. units)
10 15 20 25 30 35 40 45
Q (1/nm)
i)
ii)
iii)

Fig. S6. Simulated XRD patterns of (n, m) ice-NTs. The index (n, m) represents the chirality of the ice NTs[4]. **(a)** XRD patterns of single-shell ice-NTs with different diameters D, and illustrations of $(20,0)$ and $(14,14)$ ice-NTs as examples, where oxygen atoms in water are represented by red spheres. In ice-NTs, the peak structure appears around a Q = 20–25 (1/nm) region, which is different from the experimentally observed patterns. **(b)** Simulated XRD patterns for i) an empty-SWCNT with D = 2.4 nm, ii) a triple-walled ice NT inside the SWCNT, and iii) their difference. The chiralities for the outer, middle, and inner walls of the ice NT are (17, 10), (10, 8), and (6, 1), respectively. iv) An XRD pattern for triple-walled ice NT without the SWCNT is also shown for comparison. The right panels show the top views of the simulated systems. The oxygen atoms are represented by blue, yellow, and red spheres for each layer.

References

1. Nakai, Y. *et al*. Observation of the intrinsic magnetic susceptibility of highly purified single-wall carbon nanotubes. *Phys. Rev. B* **92,** 041402 (2015).
2. Kyakuno, H. *et al*. Diameter-dependent hydrophobicity in carbon nanotubes. *J. Chem. Phys.* **145**, 064514 (2016).
3. Kim, C. U. *et al*. Evidence for liquid water during the high-density to low-density amorphous ice transition. *Proc. Natl. Acad. Sci. USA* **106,** 4596-4600 (2009).
4. Takaiwa, D., Hatano, I., Koga, K. & Tanaka, H. Phase diagram of water in carbon nanotubes. *Proc. Natl. Acad. Sci. USA* **105,** 39-43 (2008).
